# Structural changes in calcium silicate hydrate gel and resulting improvement in phosphate species removal properties after mechanochemical treatment

**DOI:** 10.1098/rsos.181403

**Published:** 2018-12-12

**Authors:** Hirotaka Maeda, Satoshi Yokota, Toshihiro Kasuga

**Affiliations:** Department of Life Science and Applied Chemistry, Nagoya Institute of Technology, Gokiso-cho, Showa-ku, Nagoya 466-8555, Japan

**Keywords:** calcium silicate hydrate gel, planetary ball milling, phosphate species recovery, water remediation

## Abstract

The discharge of phosphate species into aqueous environments is a key issue for eutrophication prevention. In this study, we investigate a mechanochemical treatment of calcium silicate hydrate (C-S-H) gel with different organic solvents with the aim of changing its structure and improving its phosphate species removal properties. The treatment leads to a collapse of the gel structure, resulting in the formation of defective structures in the silicate anion chains. The C-S-H gel sample milled with acetone exhibits better phosphate species recovery characteristics than does the unmilled C-S-H gel sample or the C-S-H gel sample milled with 1-propanol. Ultraviolet irradiation during phosphate recovery using the C-S-H gel sample milled with acetone further enhances the recovery properties.

## 1. Introduction

The element phosphorus is an essential nutrient for the growth of microorganisms. However, the existence of large amounts of phosphate species in water is one of the main causes of eutrophication. Thus, the discharge of phosphate species in water is a major environmental concern. Conventional techniques for the recovery of phosphate species from effluents include physical processes, chemical precipitation and biological techniques [1,2]. However, the chemical precipitation method is the most widely used one owing to its low cost and because the precipitated materials can be used as fertilizers. Materials containing magnesium [3], calcium [4], iron [5] or aluminium [6] have been used as the matrix for reactions with the phosphate species for removing them. For instance, calcium silicate hydrate (C-S-H) has been used as a seed crystal for recovering phosphates from wastewater [7]. In the case of crystalline C-S-H, the high concentration of calcium ions released enhances the phosphate species recovery characteristics [8]. Porous C-S-H, which exhibits stronger capacity of calcium ion release, has been also reported to tend to have higher phosphate species recovery performance [9]. C-S-H gel is known to be metastable with respect to crystalline C-S-H, such as xonotolite and tobermorite. It is supposed that C-S-H gel has higher calcium ion releasing ability due to the higher solubility, compared with crystalline C-S-H. Moreover, C-S-H gel has been reported to exhibit better settleability, filterability and dewaterability than phosphorus precipitated with conventional CaCl_2_ and Ca(OH)_2_ [10]. C-S-H gel has great potential for use as a phosphate species recovery material. On the other hand, phosphate species removal efficiency with C-S-H gel was slightly lower than that with Ca(OH)_2_ [11].

Removing phosphate species from aqueous solutions efficiently using C-S-H gel will be a key factor for water remediation. The primary unit of C-S-H gel is a monosilicate anion structure linked by calcium polyhedral layers [12]. A nanostructure model of C-S-H gel has been proposed wherein globules of the C-S-H clusters are polydispersed at the nanoscale, resulting in the formation of fractal structures [13]. An increase in the amount of calcium ions released from C-S-H gel is expected to result in a collapse of this structure. The planetary ball milling of silica glass induces structural changes, including a breakdown of the Si–O bonds, leading to a decrease in the degree of distortion of the SiO_4_ tetrahedra [14]. We speculate that such a milling process would induce changes in the structure of C-S-H gel as well. In the present work, C-S-H gel was mechanochemically treated in order to modify its structure and hence improve its phosphate species recovery properties.

## 2. Material and methods

First, C-S-H gel samples were prepared by the hydrothermal method. In brief, a slurry with a Ca/Si molar ratio of 1.0 and consisting of silica gel, calcium hydroxide and distilled water as the solvent was hydrothermally treated at 120°C for 6 h. The liquid/solid ratio of the slurry was 25. Calcium hydroxide was prepared from the reaction of calcium oxide synthesized with heat treatment at 1000°C for 3 h of calcium carbonate. The obtained C-S-H gel samples were subjected to planetary ball milling at 600 r.p.m. for 1 h using zirconia balls with a diameter of 1 mm. Preliminary experiments performed using acetone and different rotation rates (200, 400 and 600 r.p.m.) had indicated that the structure of C-S-H gel changes dramatically after milling at 600 r.p.m. This determined the choice of 600 r.p.m. as the milling rotation rate for the rest of the experiments. The C-S-H gel/solvent/zirconia ball mass ratio was 1 : 6 : 25. It is well known that calcium carbonate is formed when C-S-H gel is soaked in distilled water. Acetone and 1-propanol, which exhibit similar relative permittivities, were used as the solvents. After the milling process, the samples were separated by filtration and dried at 80°C.

The amount of C-S-H gel in the samples was analysed by X-ray diffraction (XRD) measurements performed using a semiquantitative technique while employing silicon as an internal standard. The morphologies and chemical compositions of the samples were observed by scanning electron microscopy (SEM) and energy-dispersive X-ray spectrometry (EDS). The structural changes induced in the samples were analysed by ultraviolet/visible (UV/vis) spectroscopy performed using the diffuse reflection method with an integrating sphere and by Fourier transform infrared (FT-IR) spectroscopy performed using the KBr method. Phosphate species recovery tests were performed by the batch method while using a phosphate buffer solution with an initial concentration of 200 ppm as the model phosphate resource at 25°C. The tests were performed at a pH of 7.4 and involved shaking for 3 h. During the test, 30 mg of the test sample was soaked in 20 ml of the phosphate solution. The concentration of the phosphate species in the solution after the test was evaluated by inductively coupled plasma atomic emission spectrometry.

## 3. Results and discussion

**Figure 1.**
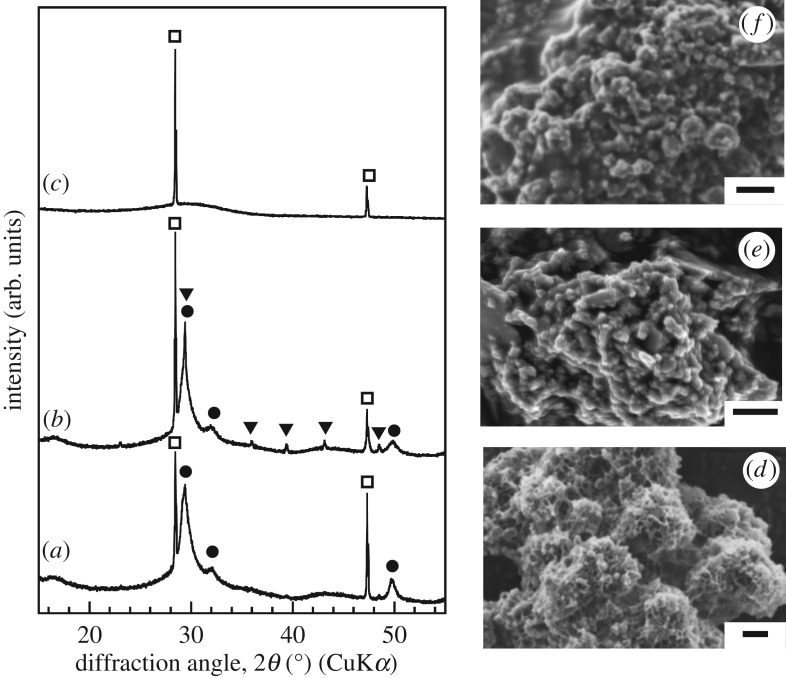
XRD patterns and SEM images of samples (*a,d*) before and after milling treatment with (*b,e*) 1-propanol and (*c,f*) acetone. Circle, C-S-H gel; triangle, calcite; square, silicon. Bar scale is 2 µm.

Figure 1 shows the XRD patterns and SEM micrographs of the various samples. Only peaks corresponding to C-S-H gel are present in the XRD pattern of the unmilled sample. On the other hand, new peaks corresponding to calcite are visible in addition to the peaks related to C-S-H gel after milling with 1-propanol. In the XRD pattern of the sample treated with acetone, there are no peaks corresponding to C-S-H gel. The amount of C-S-H gel in the samples was estimated based on the ratio of integrals of the peaks at 50° from the C-S-H gel and at 28° from the silicon. The amount of C-S-H gel present decreased after milling. This means that the milling process causes a collapse of the fractal structure of C-S-H gel. The sample milled with 1-propanol had a greater amount of C-S-H gel than did the sample milled with acetone. The zeta potentials of the samples milled with 1-propanol and acetone were determined to be approximately 31 and 0 mV, respectively. These measurements were performed using a laser Doppler velocimeter. The degree of aggregation of the samples in the two solvents affects the mechanochemical reaction, leading to differences in the changes induced in their structures. After the milling process, round particles were observed in the SEM micrographs of the two milled samples. This was in contrast to the case for the unmilled sample. The EDS analysis results showed that all the samples had a Ca/Si molar ratio of 1.0. C-S-H gel has a specific surface area of 71 m^2^ g^−1^ as determined by nitrogen gas sorption analysis. The C-S-H gel samples milled with 1-propanol and acetone had specific surface areas of 31 and 12 m^2^ g^−1^, respectively. It has been reported that, for composites containing C-S-H gel, the amount of C-S-H gel increases with an increase in the specific surface area [15]. The specific surface area of the samples decreased dramatically owing to the changes induced in their surface morphologies by milling, leading to decreases in their C-S-H gel content.

**Figure 2.**
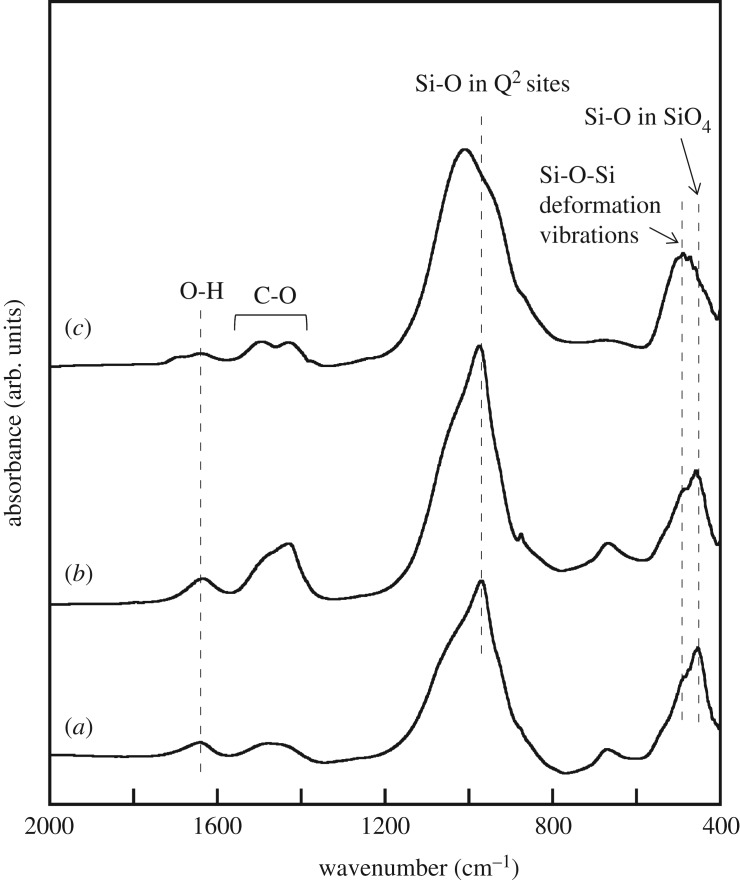
FT-IR spectra of samples (*a*) before and after milling treatment with (*b*) 1-propanol and (*c*) acetone.

Figure 2 shows the FT-IR spectra of the samples. In the spectrum of the unmilled sample, bands can be observed at approximately 460, 490 and 970 cm^−1^ along with a shoulder at approximately 1050 cm^−1^; these can be attributed to the Si–O bond in the SiO_4_ tetrahedra, the Si–O–Si bond deformation vibrations, and the Si–O bond at the Q^2^ sites of C-S-H gel, respectively [16–18]. In addition, two bands can be observed at approximately 1400–1600 and 1650 cm^−1^, which are ascribable to the C–O bond in the carbonate ions and the O–H bond in the water molecules. The spectrum of the sample milled with 1-propanol is similar to that before the milling process. This implies that milling with 1-propanol has almost no effect on the bond structure. On the other hand, milling with acetone led to changes in the bands attributable to the silicate units, including a decrease in the intensity of the band at approximately 970 cm^−1^. This band at approximately 970 cm^−1^ has been reported to be related to the structural order of C-S-H gel [19]. Thus, it can be concluded that milling with acetone promotes the deformation of the silicate units in C-S-H gel, resulting in the formation of a short-chain anion structure.

**Figure 3.**
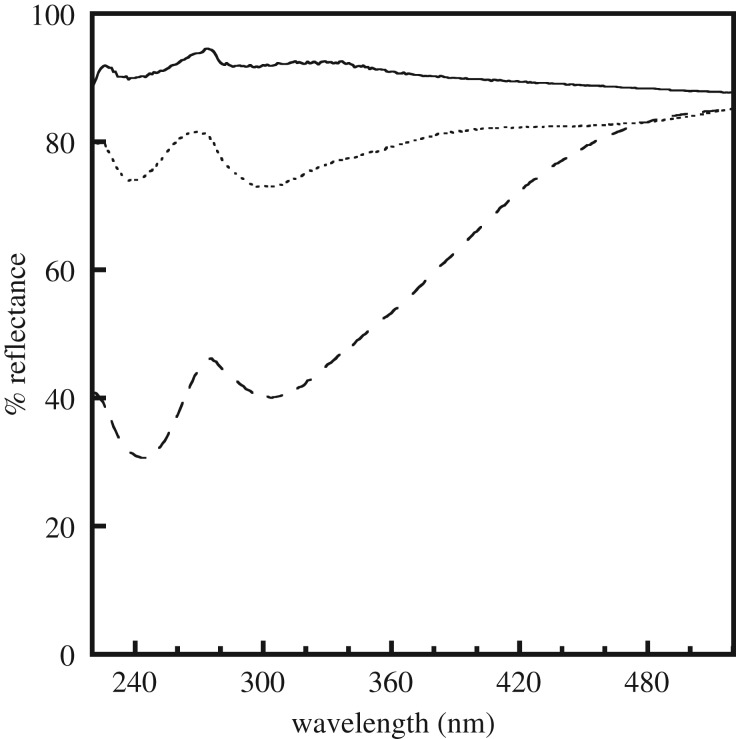
Diffuse reflectance UV/vis spectra of samples before (solid line) and after milling treatment with 1-propanol (dotted line) and acetone (dashed line).

Figure 3 shows the UV/vis spectra of the samples. No absorption peaks are present in the spectrum of the unmilled sample. On the other hand, two absorption peaks, one at approximately 245 nm and the other at 305 nm, appear after milling. The intensities of the absorption peaks in the spectrum of the sample milled with acetone are higher than those of the peaks of the sample milled with 1-propanol. The absorption peak at approximately 248 nm has been reported to be related to the oxygen vacancies in synthetic silica [20]. To our knowledge, there is no information available in the literature on the absorption peak at approximately 305 nm in the case of silicate materials. There was reported to be structural defects in the silicate chains of the C-S-H gel [21,22]. It is likely that the absorption peaks at approximately 245 and 305 nm are caused by the oxygen vacancies and defects related to the silicate chains, respectively. There were no bands, such as those attributed to a C–H bond, originating from acetone and 1-propanaol in the FT-IR spectra of the milled samples (figure 2). C-S-H gel would not react chemically with the solvent in the milling. These imply that the milling process causes microscopic changes in the silicate structure. The effect of the energy of the balls used during the milling process depends on the rotation conditions [23]. For instance, the aggregation of the sample being milled in a solvent may affect the impact energy during milling, resulting in an increase in the number of defective structures (electronic supplementary material, figure SI-1 shows the schematic structure of samples before and after the milling).

**Figure 4.**
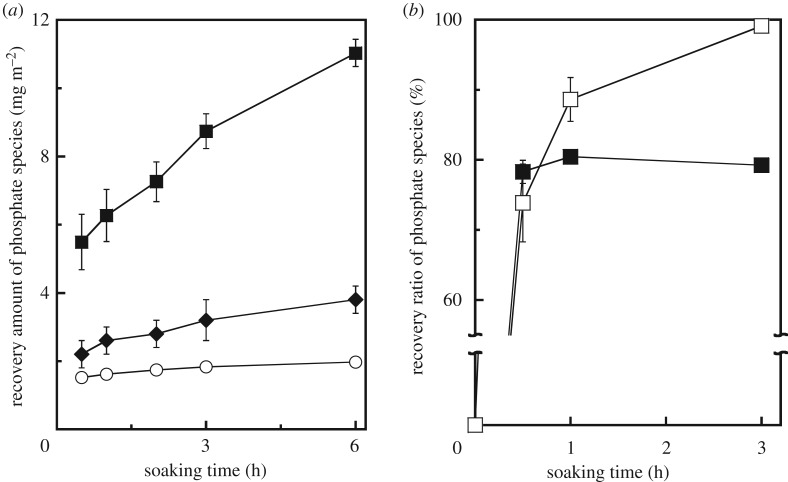
(*a*) Recovery amount of phosphate species by samples (open circle) before and after milling treatment with (diamond) 1-propanol and (filled square) acetone. (*b*) Recovery ratio of phosphate species by C-S-H gel samples after ball milling with (open square) and without (filled square) UV irradiation.

To determine the effect of the structural changes induced in the C-S-H gel samples on their phosphate species recovery properties, phosphate species recovery tests were performed using 50, 100 and 200 mg l^−1^ of a phosphate buffer solution and 1.5 g l^−1^ of the adsorbent. The equilibrium data did not fit the Langmuir and Freundlich models for any of the samples. This suggests that the recovery of phosphate species using C-S-H gel does not occur through monolayer adsorption on the samples but through the precipitation of the phosphate minerals in the solution. The recovered amounts of the phosphate species were calculated by dividing the concentration of the phosphate species in the test solution by the specific surface areas of the samples; this was done to elucidate the interactions between the phosphate species and the calcium and silicate ions generated by the dissolution of the samples in the solution, as shown in figure 4*a*. It was observed that the milling process enhances the phosphate species recovery properties. Furthermore, the sample milled with acetone exhibited better recovery properties than did the sample milled using 1-propanol. EDS analysis results indicated that, after the recovery test, all the samples contained P as well as Si and Ca (P/(Si + Ca) = 0.5–0.8). On the other hand, Ca ions were not detected in the solution when the unmilled and milled samples were soaked in it. In preliminary experiments, the sample milled with acetone had exhibited a stronger Ca-ion-releasing ability (3.0 mg m^−2^) than those of the unmilled sample (0.3 mg m^−2^) and that milled with 1-propanol (1.8 mg m^−2^) when the samples had been soaked in distilled water for 30 min. On the other hand, the concentrations of silicate species in the solutions were similar when the unmilled and milled samples were soaked in them for 6 h. These results suggest that the structural changes induced by the milling process enhanced the dissolution of the samples, resulting in the activation of precipitation through a reaction of the phosphate species with their counter cations.

To further investigate the effect of the defective structures on the phosphate species recovery properties, UV irradiation was performed during the recovery tests. The phosphate species removal properties of the samples were evaluated using a solid/liquid ratio of 1.5 g l^−1^ and an initial phosphate species solution concentration of 100 ppm. The intensity of the UV radiation at 10 cm was approximately 4.0 mW cm^−2^. Figure 4*b* shows the phosphate species removal properties of the C-S-H gel sample milled with acetone with and without UV irradiation at 25°C. The phosphate species recovery ratio increased sharply and then plateaued when UV irradiation was not performed. On the other hand, the recovery rate increased exponentially with time when UV irradiation was performed. By contrast, UV irradiation had no effect on the phosphate species removal properties of the unmilled sample. In the case of monolayer BiO_2−x_, the presence of rich vacancy promotes degradation activities for phenol and rhodamine B in aqueous solution under UV irradiation [24]. Thus, it can be concluded that the UV radiation was absorbed by the defective sites of the milled samples, resulting in an improvement in their phosphate species recovery properties.

## 4. Conclusion

C-S-H gel samples were synthesized by planetary ball milling using two organic solvents in order to investigate the effect of the milling process on their structure and hence their phosphate species recovery properties. It was found that the structural changes induced as well as the amount of C-S-H gel in the samples depend on the type of organic solvent used. The milling process affected the silicate structure in C-S-H gel, resulting in the formation of defective structures. The sample milled with acetone exhibited the strongest ability to recover phosphate species, owing to its improved solubility. Further, by combining the mechanochemical treatment of the C-S-H gel with UV irradiation during the phosphate species recovery test, the recovery characteristics could be improved further, making this process a highly suited one for the recovery of phosphate species from aqueous solutions.

## Supplementary Material

Fig SI-1

## Data accessibility

The datasets supporting this article have been uploading as part of the electronic supplementary material.
