# Supplementary material for: Structural changes in calcium silicate hydrate gel and resulting improvement in phosphate species removal properties after mechanochemical treatment
Source: R Soc Open Sci. 2018 Dec 12;5(12):181403. doi: 10.1098/rsos.181403 (PMC6304137; doi:10.1098/rsos.181403)
Supplement: Fig SI-1 [file rsos181403supp1.pptx]

## Slide 1
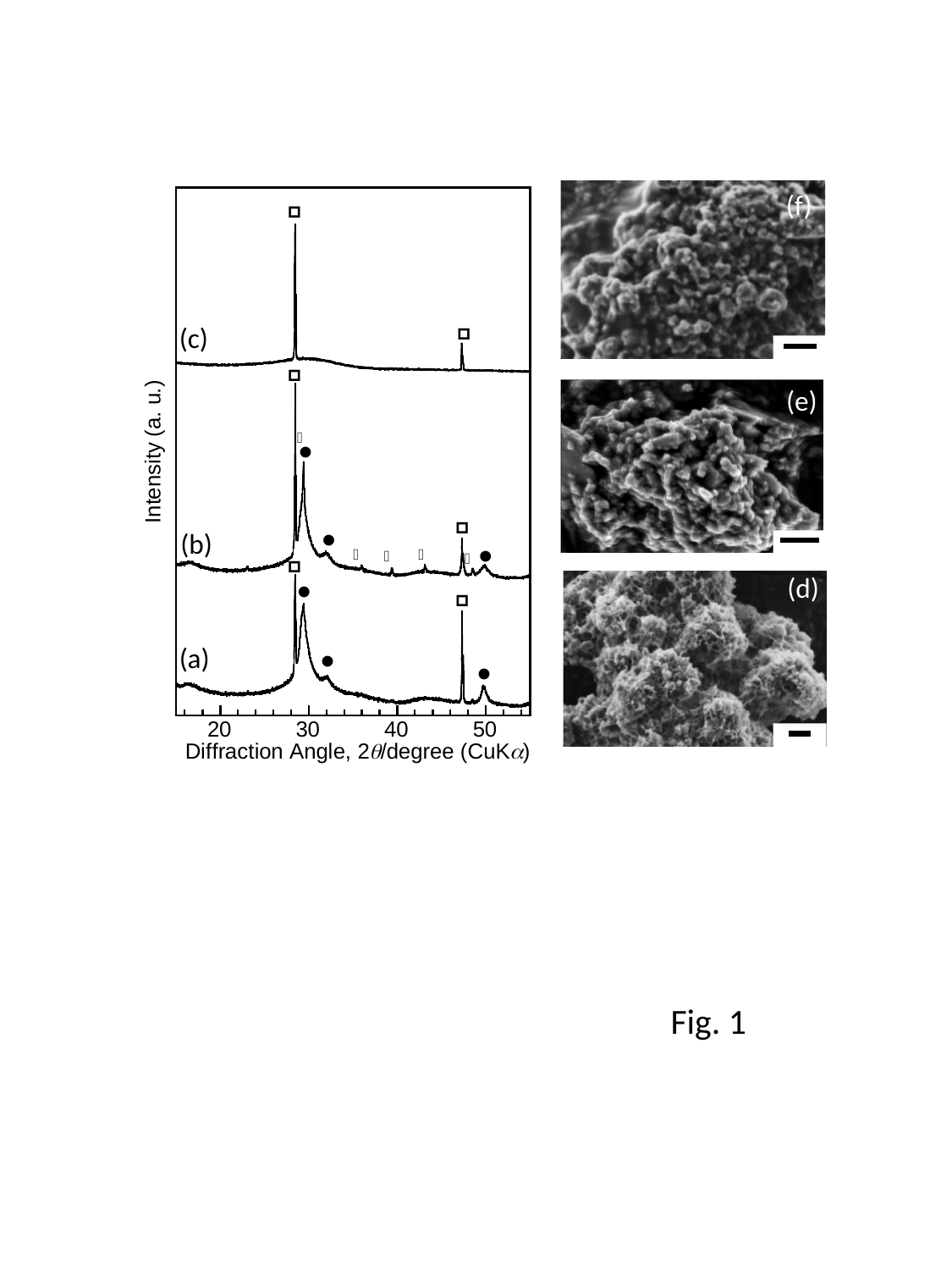

(f)
(c)
(e)
(b)
(d)
(a)
Fig. 1

## Slide 2
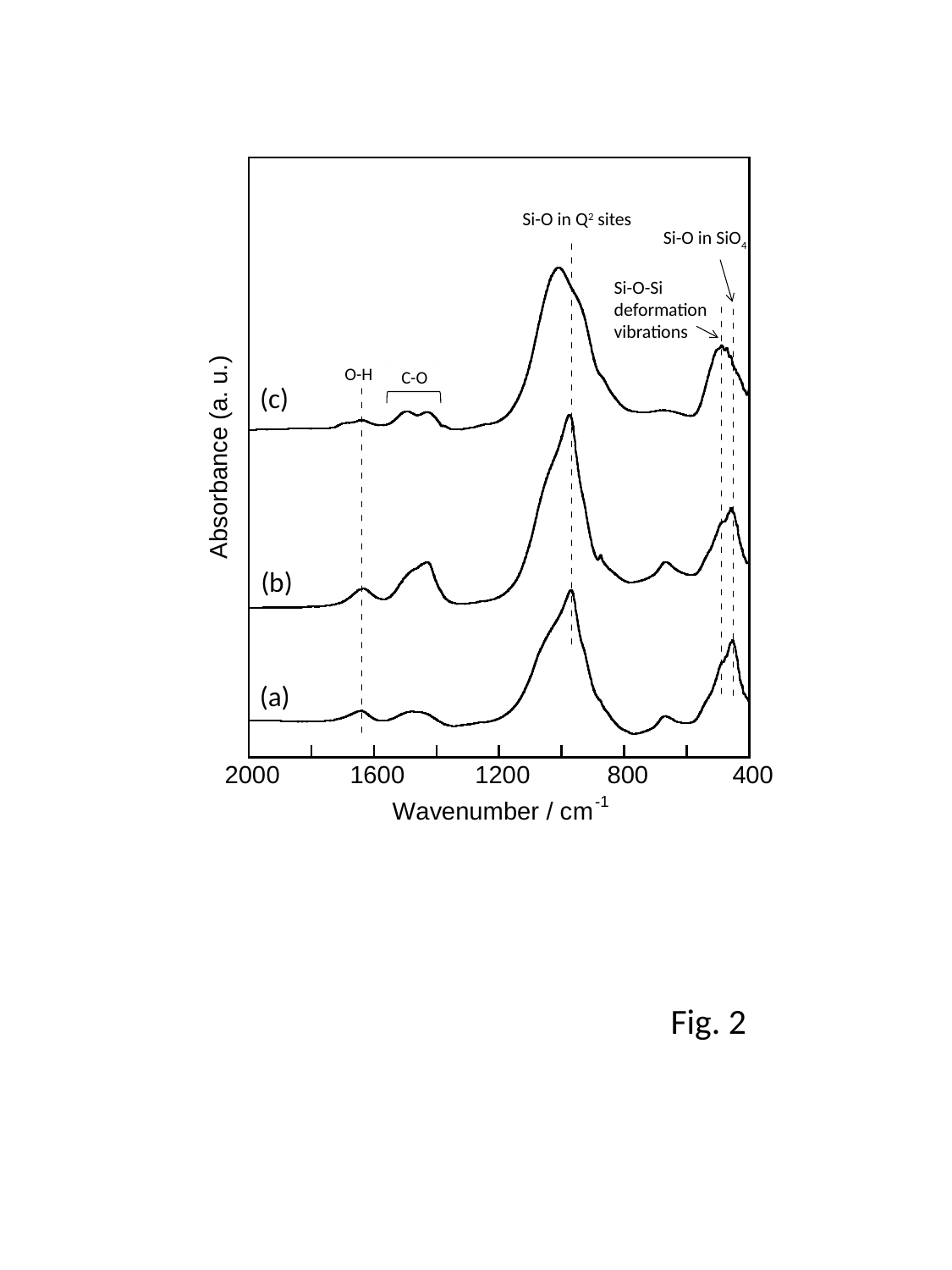

Si-O in Q2 sites
Si-O in SiO4
Si-O-Si
deformation vibrations
O-H
C-O
(c)
(b)
(a)
Fig. 2

## Slide 3
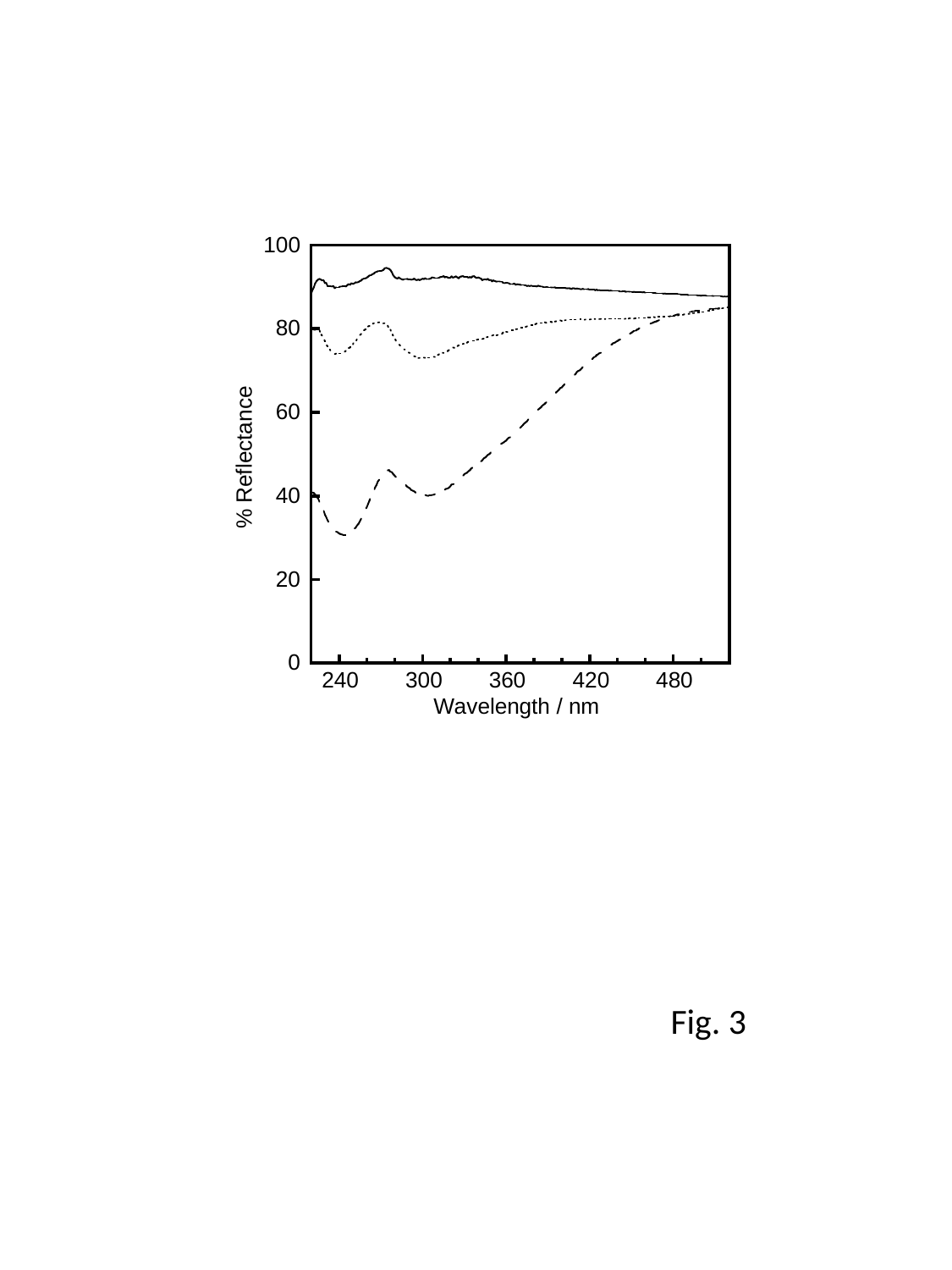

Fig. 3

## Slide 4
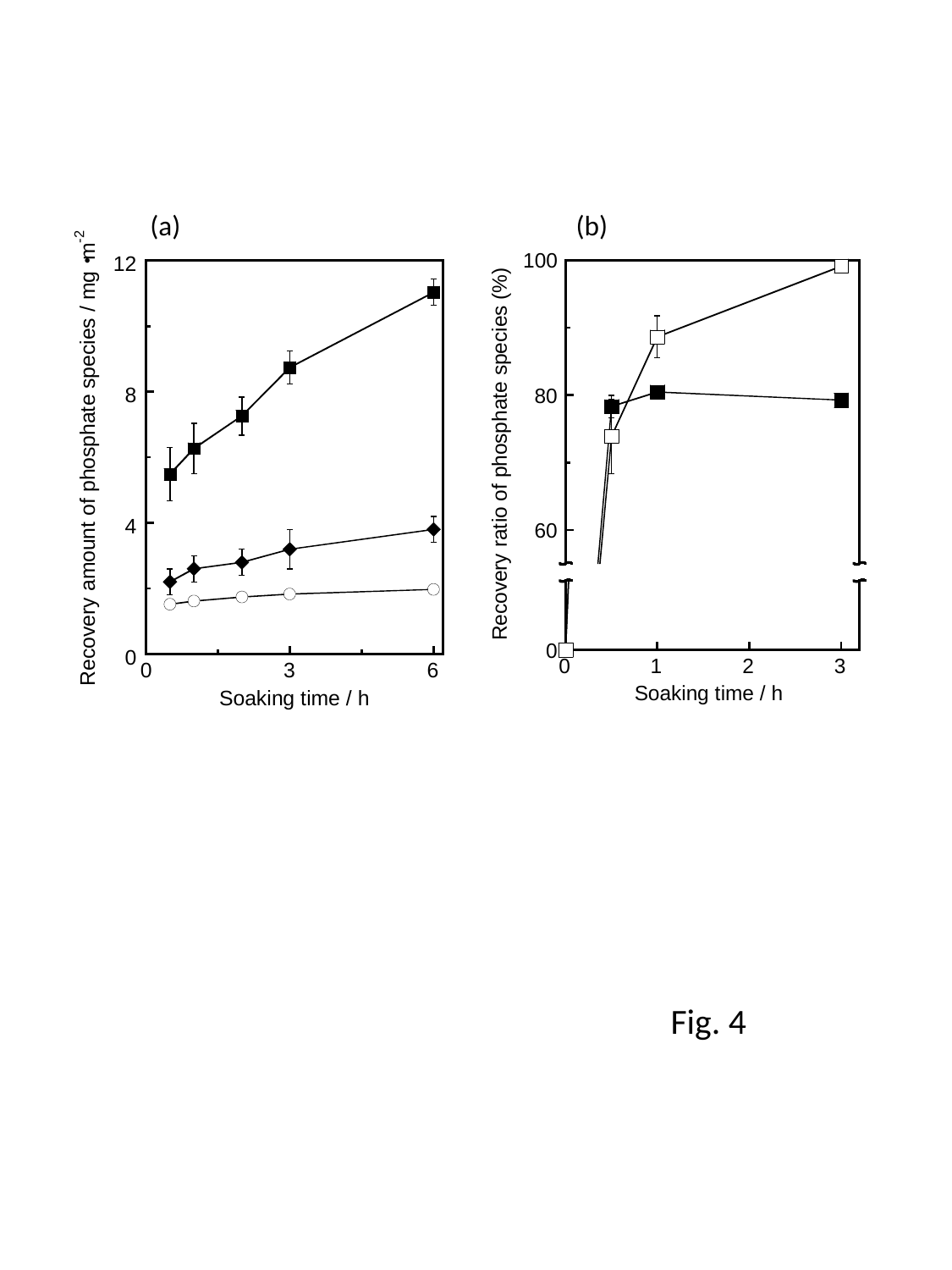

(a)
(b)
Fig. 4

## Slide 5
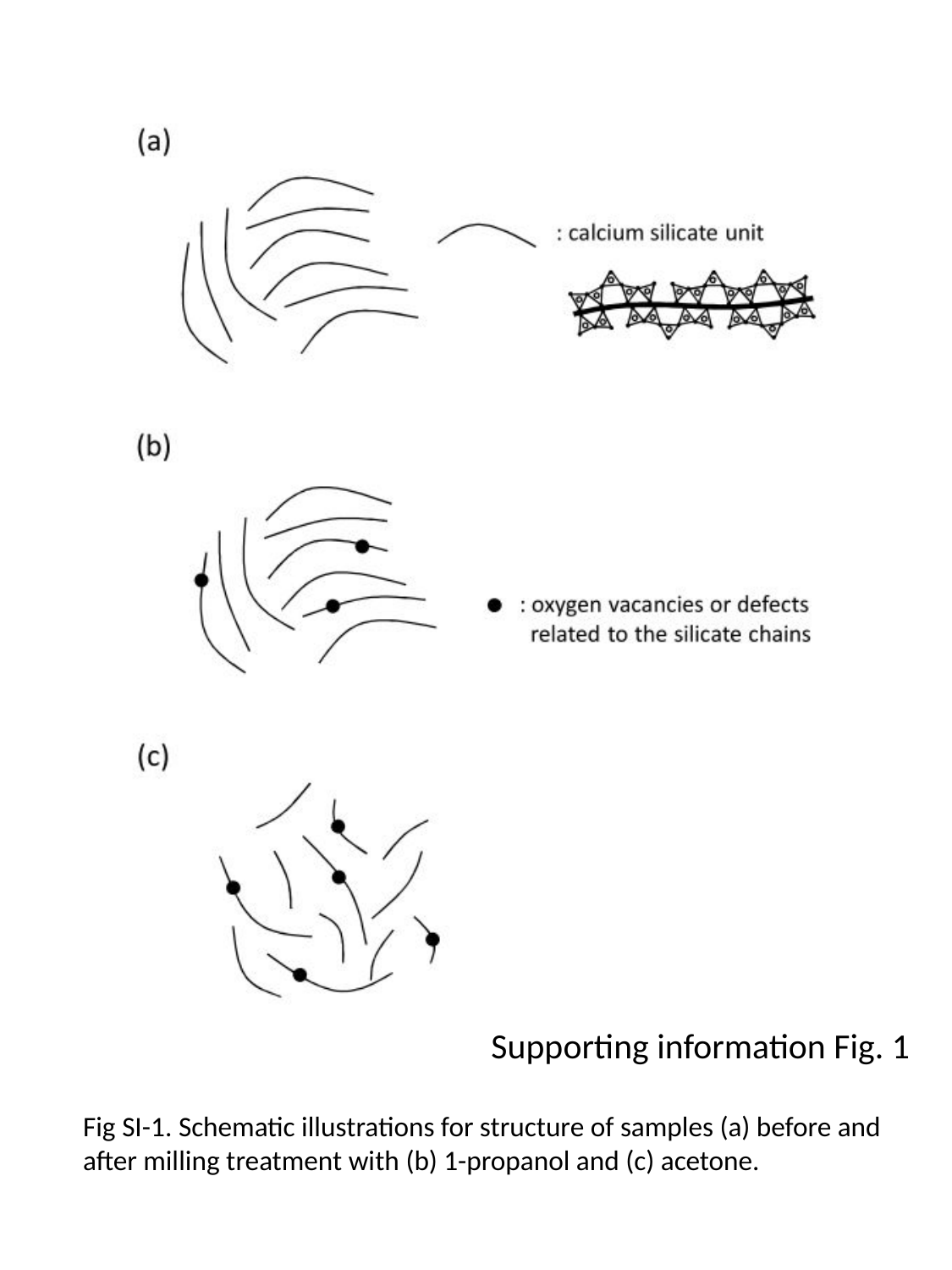

Supporting information Fig. 1
Fig SI-1. Schematic illustrations for structure of samples (a) before and after milling treatment with (b) 1-propanol and (c) acetone.
